# Observation of a Two-Dimensional Hydrophobic Collapse at the Surface of Water Using Heterodyne-Detected Surface Sum-Frequency Generation

**DOI:** 10.1021/acs.jpclett.3c01530

**Published:** 2023-10-10

**Authors:** Sanghamitra Sengupta, Jan Versluis, Huib J. Bakker

**Affiliations:** AMOLF, Science Park 104, 1098 XG Amsterdam, The Netherlands

## Abstract

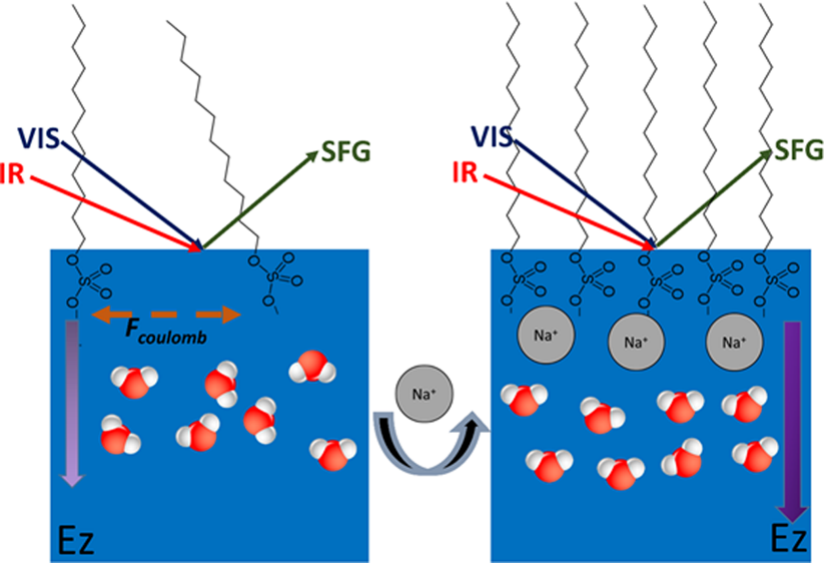

We study the effect of sodium chloride (NaCl) on the properties of the interface of water and the surfactant dodecyl sulfate (DS^–^) using heterodyne-detected vibrational sum-frequency generation spectroscopy. We find that the signal of the O–H stretch vibrations of oriented water molecules at the interface is highly nonlinearly dependent on the NaCl concentration. This nonlinear dependence is explained by a combination of screening of the electric field of surface-bound DS^–^ ions pointing into the bulk and screening of the Coulomb repulsion between the headgroups of the DS^–^ ions in the surface plane. The latter effect strongly increases the oriented water signal within a limited NaCl concentration range of 10–100 mM, indicating a two-dimensional hydrophobic collapse of the surfactant layer. The occurrence of collapse is supported by model calculations of the surface potential and surface surfactant density.

Surfactant-encapsulated macromolecular ensembles attract a great deal of research interest due to their widespread application in the field of improved and sustainable electronic devices^1−3^ and protein denaturation^4−7^ processes. Sodium dodecyl sulfate (SDS) is one of the most extensively used surfactants for these purposes, comprising a hydrophobic tail and a singly negatively charged headgroup. Interestingly, the effects of SDS on macromolecular systems strongly depend on the local ionic environment; for instance, the addition of external electrolytes was found to affect its efficiency in protein denaturation^4,8^ and in the separation of single-walled carbon nanotubes.^9,10^ Many experimental^11,12^ and theoretical^13,14^ studies have attempted to unravel this effect, but a molecular-level picture is still missing. Current research efforts primarily involve bulk measurements,^15,16^ mostly due to a lack of surface-specific and surface-sensitive techniques that can be performed under ambient conditions.

Surfactants are mostly studied at concentrations in the vicinity of their critical micellar concentration (CMC), leaving the properties of lower SDS concentrations fairly unexplored. It is well established in the literature that the addition of salts decreases the CMC of SDS drastically.^17^ The CMC of SDS is 8 mM in bulk, and the results show that the addition of salts can decrease the CMC of SDS to nearly 1 mM. Low concentrations of SDS are highly relevant for its biofunctional properties. Recent research work showed that a low concentration of SDS can already induce protein denaturation^18−21^ and other structural changes by complexing with proteins,^7^ even in biogeochemical processes.^22^ Unfortunately, few techniques enable the study of the formation of these complex macromolecular assemblies at the molecular level. Given the importance of these assemblies, molecular-level information is urgently needed and would be immediately beneficial. This is a multifold challenge as different interactions are involved: (i) ionic interaction with SDS, (ii) interaction of SDS with proteins or nanotubes, and (iii) ion-specific effects on the properties of the macromolecules.

In this work, we examine the interaction between SDS and NaCl at different SDS and NaCl bulk concentrations using heterodyne-detected vibrational sum-frequency generation spectroscopy^23,24^ (HD-VSFG) at the liquid–air interface. HD-VSFG is a surface-specific technique that is capable of capturing vibrational features from molecular subgroups where the inversion symmetry is broken and the net dipolar contribution is non-zero. The details of the experimental setup specific to these experiments can be found elsewhere.^25^ We investigated solutions with SDS concentrations ranging from 10 to 100 μM and by varying the NaCl concentration from 0 mM to 1 M. For all studied SDS concentrations, we observe a highly nonlinear dependence of the water signal on the NaCl concentration detected with HD-VSFG, which indicates the occurrence of a two-dimensional (2D) hydrophobic collapse of the surfactant layer in a limited NaCl concentration range of 10–100 mM. We complemented the experimental data with theoretical modeling based on the Poisson–Boltzmann theory, which supports the occurrence of a 2D phase transition beyond a NaCl concentration of ∼10 mM.

Figure 1 shows HD-VSFG spectra of aqueous solutions containing 25 μM SDS and different NaCl concentrations ranging from 10 mM to 1 M. We performed the same NaCl concentration series experiments for 10, 25, 50, and 75 μM SDS solutions. The measured HD-VSFG spectra of those series can be found in SI 1.

**Figure 1:**
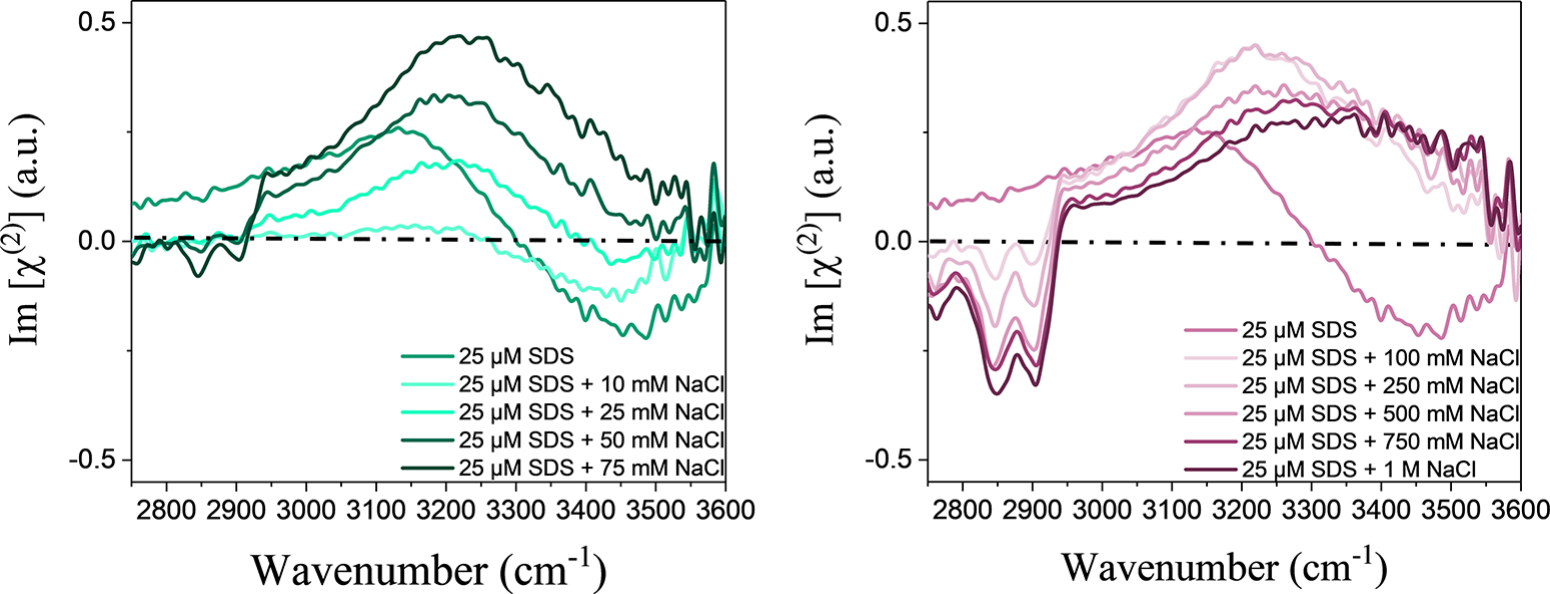
Heterodyne-detected vibrational sum-frequency generation (HD-VSFG) spectra of a solution of 25 mM sodium dodecyl sulfate (SDS) and different concentrations of sodium chloride (NaCl). The left panel lists the lower NaCl concentrations, and the right panel the higher NaCl concentrations.

In the frequency region of 2800–3000 cm^–1^, we observe three distinct vibrational features associated with the C–H vibrations of the hydrophobic tail of the DS^–^: a negative band at 2850 cm^–1^ that has a contribution from both the symmetric stretch of the methylene (CH_2_) and the terminal methyl (CH_3_) groups of the hydrophobic tail of DS^–^, a second negative band at 2930 cm^–1^ assigned to the Fermi resonance between the CH_3_ symmetric stretching and bending mode, and a positive band at 2960 cm^–1^ that we assign to the asymmetric stretch of the terminal CH_3_ group.

In the frequency region of 3000–3600 cm^–1^, we observe the signal of the O–H stretch vibrations of water molecules close to the surface. Due to the negative charge of the headgroups of the DS^–^, the water molecules are oriented with their O–H groups to the surface, giving rise to a positive Im[χ^(2)^] of the O–H stretch vibrations. For a solution containing 25 μM SDS and a low concentration of NaCl, the Im[χ^(2)^] spectrum has a negative sign at frequencies of >3300 cm^–1^. For these solutions, the surface density of DS^–^ ions is low, and the surface electric field exerted by these ions is weak. The Im[χ^(2)^] spectrum will thus be similar with the Im[χ^(2)^] spectrum of pure H_2_O that is negative in the O–H stretch vibrational region up to a frequency of 3600 cm^–1^ and has its absolute maximum at a frequency of ∼3500 cm^–1^.^24^ In addition, for a solution containing 25 μM SDS and up to a few millimolar NaCl, the Debye screening length is long and the SFG signal originates in large part from regions deeper down in the solution, leading to a phase distortion that enhances the negative Im[χ^(2)^] signal at frequencies of >3300 cm^–1^. For NaCl concentrations of >25 mM, the Im[χ^(2)^] spectrum is positive at all frequencies in the O–H stretch vibrational region, because the phase distortion has vanished and the surface density of DS^–^ ions has increased, as we will discuss below. For a solution of SDS at its CMC of 8 mM, the Im[χ^(2)^] spectrum is also positive at all O–H stretch frequencies (Figure SI 2), which was also found in ref (26), because at this concentration the surface density of DS^–^ is high and the Debye screening length is short.^26^

For NaCl concentrations of ≤250 mM, the shape of the O–H stretch spectrum does not change, and only the amplitude changes. For concentrations of >500 mM, the spectrum shows a slight distortion, in particular a shift to higher frequencies. In interpreting this spectral change, we follow the work of refs (27−29). In these works, it was demonstrated that the spectrum of the O–H vibrations of water underneath a (charged) surfactant layer is formed by the sum of the contribution of a bonded interface layer (BIL), representing water molecules that are directly bonded to the interface and the contribution of water molecules in the diffuse layer (DL) below the interface.^27−29^ At low salt concentrations (<250 mM), we expect the response of the DL layer to dominate, as is corroborated by the fact that the spectral shape does not change in this concentration range. We find that in this region the Im[χ^(2)^] spectrum of water is largely formed by a broad response centered at 3250 cm^–1^. The change in the spectral shape observed for NaCl concentrations of >250 mM is likely due to the increased relative contribution of the BIL response, due to the fact that the DL response will become small at these higher concentrations due to screening of the surface electric field. The BIL response will be dominantly formed by water molecules hydrogen-bonded to the sulfate groups of the DS^–^ surfactant. These hydrogen bonds will likely be somewhat weaker than those between water molecules in the bulk, thus explaining the small blue shift of the spectrum.

Figure 2 shows the amplitude of the water O–H stretch signal at 3250 cm^–1^ as a function of NaCl concentration. Many of the data points correspond to the amplitudes of the HD-VSFG spectra shown in Figure 1, but we also added the amplitudes of spectra measured at lower salt concentrations down to 10 mM. We corrected the signals at very low concentrations (<1 mM) for phase distortion effects. The water signal shows an intriguing, strongly nonlinear dependence on the salt concentrations that can be broken down into three distinct regions: (i) an initial steep decrease between 0 and 10 mM NaCl, (ii) a steep increase between 10 and 100 mM NaCl, and (iii) a gradual decrease between 100 mM and 1 M NaCl. For those hydrogen-bonded bulk water molecules, it should be noted that in salt concentration regions i and ii the water O–H stretch signal is completely dominated by the DL response, which implies that the observed nonlinear dependence of the amplitude of the signal on the salt concentration is not the result of a change of the contribution of the BIL response to the signal.

**Figure 2:**
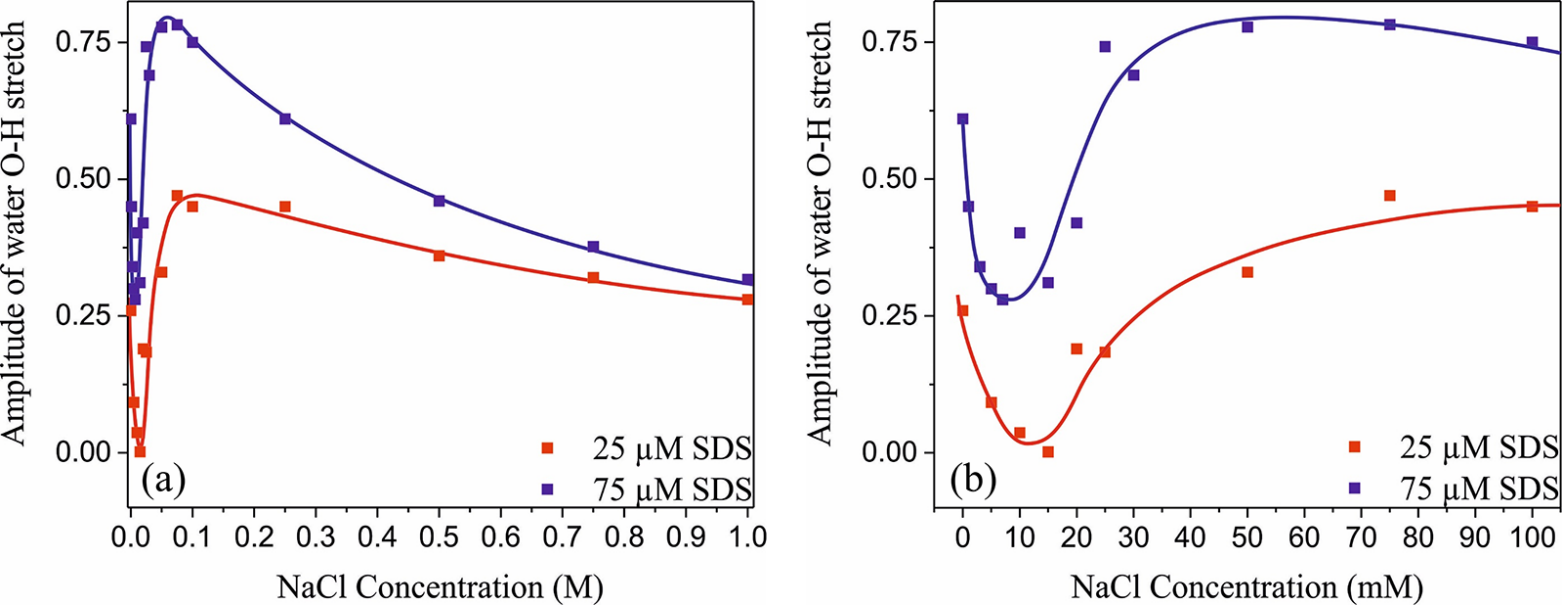
Heterodyne-detected vibrational sum-frequency generation (HD-VSFG) signal of the water O–H stretch vibrations at 3250 cm^–1^ as a function of NaCl concentration for two different SDS concentrations. The symbols represent the data points, and the lines are guides to the eye. Panel b is a close-up of the water signal between 0 and 100 mM NaCl.

The dependence of the water signal on the salt concentration can be explained by the interplay of the two effects. The first effect is the screening of the electric field of the negative headgroups into the bulk of the solutions. The added Na^+^ ions will have a screening effect and decrease the length over which this field penetrates the bulk (in the *Z* direction). As a result, water molecules will show a smaller degree of orientation with their hydrogen atoms pointing toward the surface, which leads to a decrease in the Im[χ^(2)^] response of the O–H stretch vibrations of these water molecules. This decrease in the water signal with an increase in salt concentration is very fast at low salt concentrations and becomes more gradual at higher salt concentrations. The second effect is the lateral screening of Coulomb repulsion in the plane of the surface by the added Na^+^ ions. Due to this screening, the DS^–^ ions come closer to each other, enabling a favorable short-range van der Waals interaction between the hydrophobic tails of the DS^–^ ions, as soon as their average mutual distance becomes smaller than a critical value. From 10 mM NaCl on, this favorable interaction results in a hydrophobic collapse of the DS^–^ ions at the surface, leading to a steep increase in the surface density of DS^–^ and thereby the water signal. For NaCl concentrations of >100 mM, the surface is fully covered with a monolayer of DS^–^ and the surface density will not further increase. At these higher concentrations, the screening effect of the electric field pointing into the bulk again becomes dominating, leading to a decrease in the number density of oriented water dipoles, resulting in a weaker HD-VSFG signal.^30,31^ We thus find that the addition of NaCl induces a transition from a gas-phase DS^–^ surface layer to a liquid condensed-phase surface layer. Such a transition is reminiscent of the behavior observed in surface tension isotherms of Langmuir monolayers, the difference being that for Langmuir monolayers the change in phase is induced by physical compression of the surface.^32^

Figure 3 represents the amplitude of the symmetric C–H stretch vibrations at 2850 cm^–1^ as a function of NaCl concentration. Without added salt, the C–H stretch signal is negligibly small, reflecting a very low surface concentration of DS^–^. With an increase in NaCl concentration, the C–H signal appears and then increases rapidly, reflecting a strong increase in the surface density of the DS^–^ surfactants. In addition to the increase in surface density, the amplitude of the C–H stretching bands likely also increases because of an enhanced orientation of the aliphatic tails of the DS^–^ ions perpendicular to the water–air interface.^33^ The NaCl concentration at which the C–H vibration becomes visible and starts to increase strongly depends on the SDS bulk concentration. This concentration lag decreases with an increasing SDS concentration. Thereby, these observations support the explanation that beyond a certain Na^+^ concentration, the DS^–^ ions get sufficiently close to have a highly favorable van der Waals interaction of their hydrophobic tails, leading to a 2D hydrophobic collapse. The Na^+^ concentration at which this happens decreases with an increasing bulk SDS concentration.

**Figure 3:**
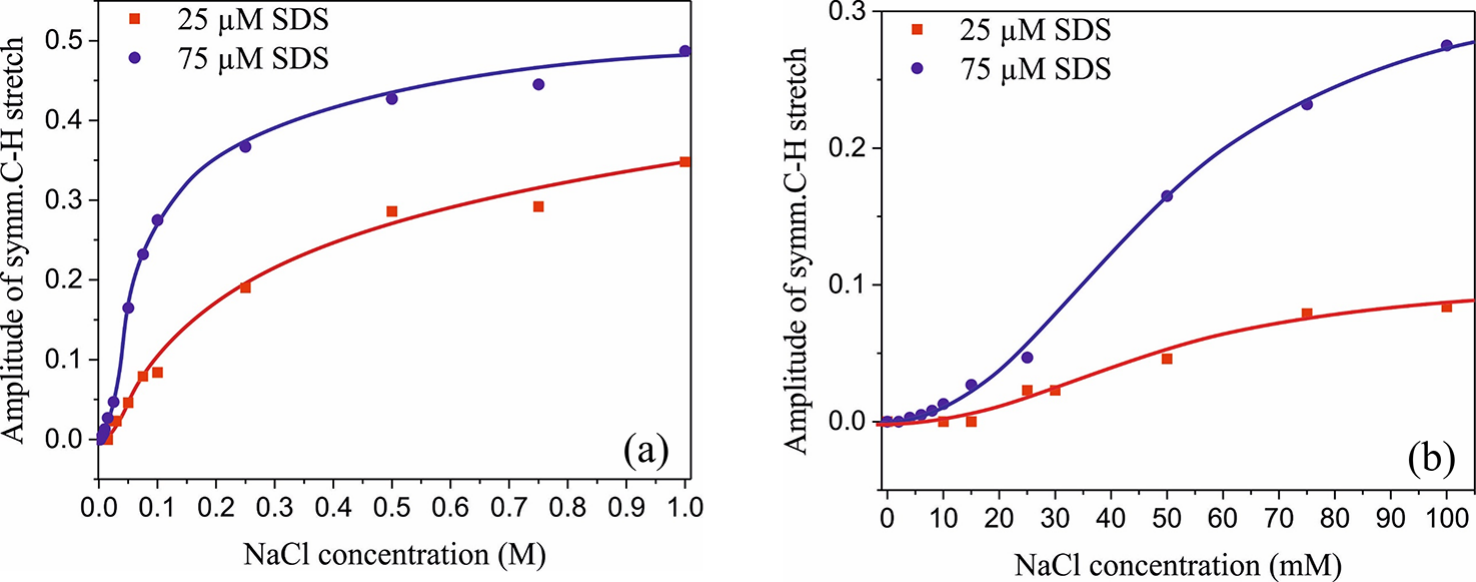
(a) Amplitude of the symmetric C–H stretch vibrational band at 2850 cm^–1^ as a function of NaCl concentration for two different SDS bulk concentrations. The markers represent the data points, and the bold lines are guides to the eye. (b) Close-up version of panel a between 0 and 100 mM NaCl.

Our experimental observations show that by tuning the ionic strength, we can dramatically change the surfactant density at the interface, tune the surface potential, and change the number density of oriented water molecules near the interface. We thus find that ions not only lead to the screening of the electric field exerted by charged surfactants into the bulk but also lead to a dramatic increase in the surface density of charged surfactants due to the screening of the lateral Coulomb repulsion between the charged surfactant headgroups within the plane of the interface. The most surprising observation is that this increase in surface density occurs in a highly limited salt concentration interval, which indicates that the surface layer undergoes a strong change in density and order when the average mutual distance between the surfactants becomes smaller than the critical value. The observations thus point to a 2D hydrophobic collapse that is enabled by the favorable short-range van der Waals interaction of the hydrophobic tails of the surfactants.

To quantify the competition between the screening of the electric field in the bulk and the screening of the Coulomb repulsion in the surface plane, we complemented our experimental observations with model calculations. In these calculations, we have modified the traditional Langmuir isotherm and coupled it to the full Poisson–Boltzmann (PB) equation^34^ where we have incorporated the SDS surface concentration dependence in calculating the free energy of adsorption of SDS at the interface. The PB equation is widely used to calculate the surface potential for chemical and biological systems.^35,36^ We made two modifications to the traditional Langmuir isotherm. The first is that we include the contribution of the surface potential to the free energy. The second modification is that we introduce a free energy gain term depending on the surface density/surface coverage of SDS molecules, representing the favorable van der Waals interaction that arises beyond a particular surface density. The detailed numerical equations used in the modeling can be found in SI 3.

The results of the calculations are shown in Figure 4a and are in excellent agreement with the experimental results, reproducing the three distinct regions for the water response. Without the extra free energy gain leading to the 2D hydrophobic collapse, the calculations do not reproduce the experimental observations, as shown in Figure 4b. The calculations also show that the surface density of SDS molecules reaches saturation faster for higher bulk SDS concentrations, in agreement with the experimental observations.

**Figure 4:**
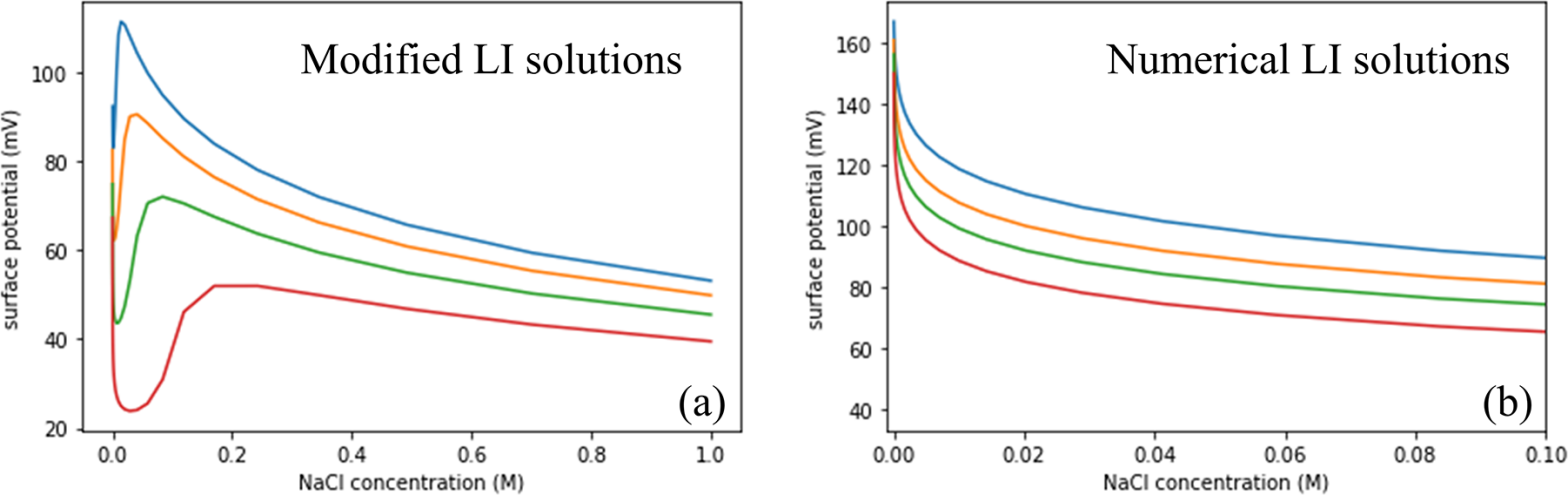
Calculation of the surface potential of an aqueous solution of SDS and NaCl as a function of NaCl bulk concentration for four different SDS concentrations: red, green, orange, and blue curves present 10 μM (red), 25 μM (green), 50 μM (orange), and 75 μM SDS (blue), respectively. In panel a, the calculated curves are obtained with a modified Langmuir isotherm (LI) that describes a favorable free energy contribution beyond a certain surface density, representing a two-dimensional hydrophobic collapse. In panel b, the curves represent calculations using the traditional LI equation. The detailed equations used for the calculations can be found in SI 3.

To further study the competition between the screening of the electric field into the bulk and the screening of the Coulomb repulsion in the surface plane, we performed similar experiments with a positively charged surfactant, dodecyltrimethylammonium bromide (DTAB). We observe the same trends in the water signal and the signal of the C–H stretch vibrations as a function of the increasing NaCl concentration. The imaginary spectra of solutions of 75 μM DTAB and different concentrations of NaCl can be found in SI 4. We thus conclude that the competition between the screening of the electric field into the bulk and the screening of the Coulomb repulsion in the surface plane is a general effect for charged surfactants.

In summary, the HD-VSFG studies presented here provide a deeper understanding of the hydrophobic and electrostatic interactions of charged surfactants and how these interactions are influenced by added ions. These observations are highly relevant for the understanding of how surfactants and added ions affect the conformation of macromolecular systems (e.g., proteins). This knowledge will be extremely beneficial for structural biology and the understanding of the properties and functioning of biological membranes and protein denaturation mechanisms.

## Supporting Information Available

HD-VSFG spectra of four different SDS concentrations each at different NaCl concentrations, HD-VSFG spectra of SDS at the CMC at different NaCl concentrations, modified Langmuir isotherm model used in this study, HD-VSFG spectra of 75 μM DTAB at different NaCl concentrations (PDF)
Python code for calculation (PDF)
Transparent Peer Review report available (PDF)
