# Supplementary material for: Observation of a Two-Dimensional Hydrophobic Collapse at the Surface of Water Using Heterodyne-Detected Surface Sum-Frequency Generation
Source: J Phys Chem Lett. 2023 Oct 10;14(41):9285–90. doi: 10.1021/acs.jpclett.3c01530 (PMC10591499; doi:10.1021/acs.jpclett.3c01530)
Supplement: Supplementary file 1 — jz3c01530_si_001.pdf [file jz3c01530_si_001.pdf]

# Observation of a Two-Dimensional Hydrophobic Collapse at the Surface of the Water using Heterodyne-Detected Surface Sum-Frequency Generation

Sanghamitra Sengupta[*†], Jan Versluis[†], and Huib J. Bakker[†]
AMOLF, Science Park 104, 1098 XG, Amsterdam, The Netherlands
Corresponding author's email: s.sengupta@amolf.nl

## Supplementary information:

(i) HDVSFG spectra of four different SDS concentrations each at different NaCl concentrations.
(ii) HDVSFG spectra of SDS at the critical micellar concentration (CMC) at different NaCl concentrations.
(iii) Modified Langmuir isotherm model used in this study.
(iv) HDVSFG spectra of 75 μM Dodecyltrimethylammonium bromide (DTAB) at different sodium chloride (NaCl) concentrations.

**SI 1: HDVSFG spectra of four different SDS concentrations each at different NaCl concentrations.**

Below we show the HDVSFG spectra of different SDS concentrations at a range of NaCl concentrations. The NaCl concentrations remain the same for all SDS concentrations. Except for 10 µM SDS, the other three concentrations show the same trend in overall behavior as mentioned in the main text. In the left panel, we have lower NaCl concentrations and in the right panel, we have higher NaCl concentrations.

**10 µM SDS**

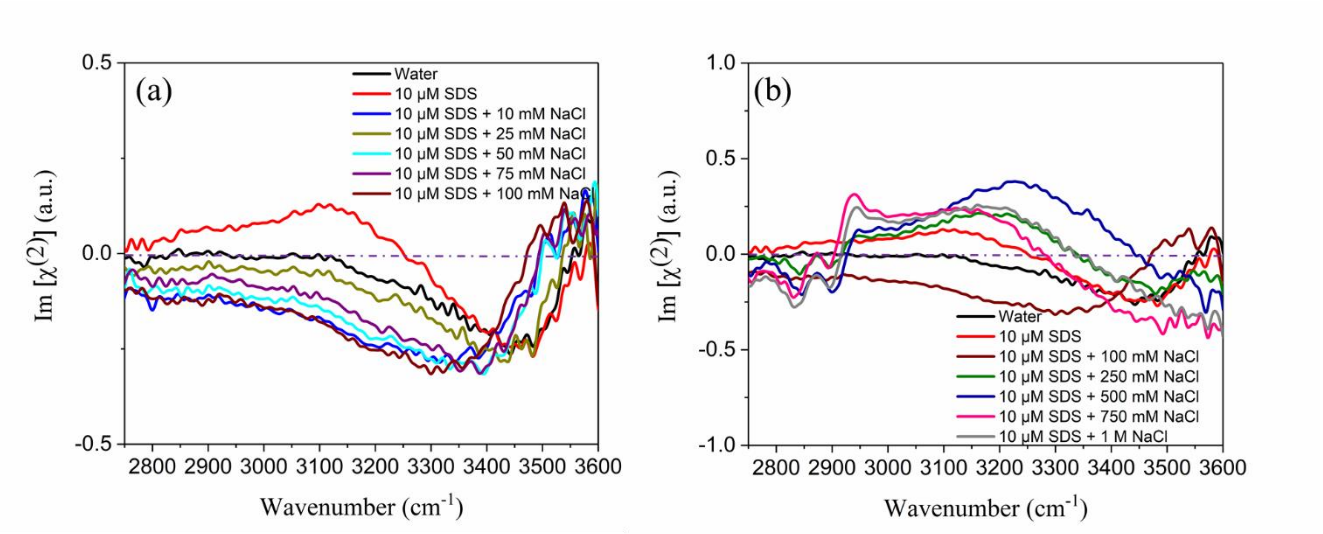


**25 µM SDS**

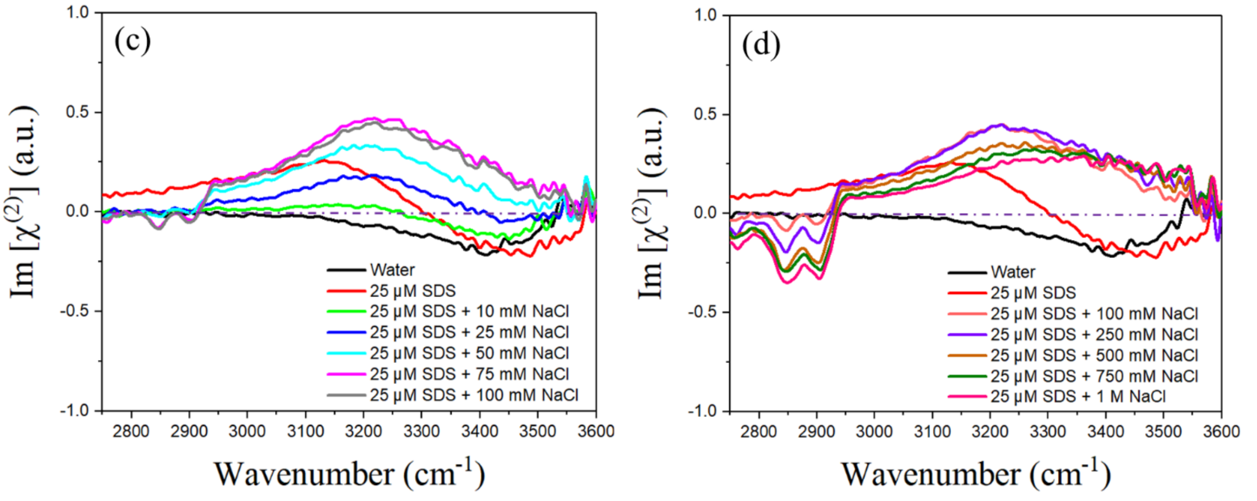


**50 µM SDS**

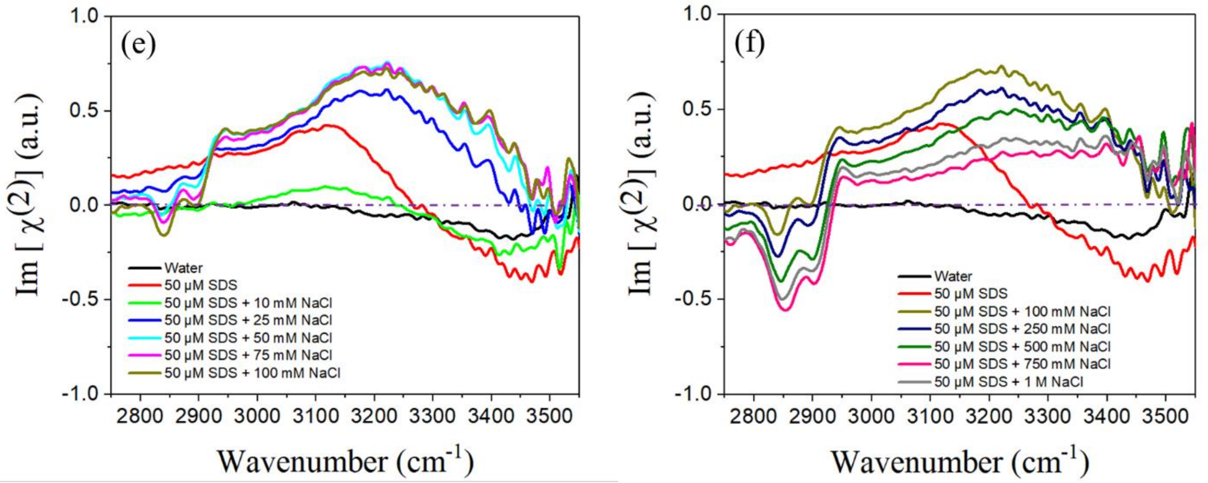


## 75 µM

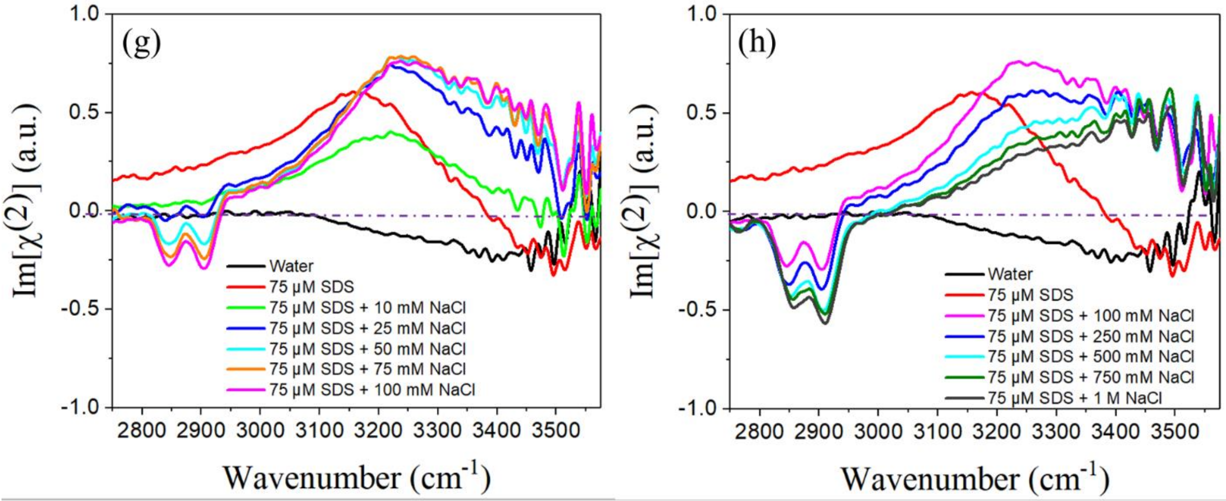


**Figure 1**: This figure shows the HDVSFG spectra of 10 µM, 25 µM, 50 µM, and 75 µM SDS concentrations at different NaCl concentrations. Figures a,c,e, and g show the features from the lower NaCl concentrations, and b,d,f, and h show the features from higher NaCl concentrations.

## SI 2: HDVSFG spectra of SDS at a critical micellar concentration (CMC) at different NaCl concentrations.

The spectra below show the HDVSFG spectra of SDS at its CMC with the addition of different sodium chloride (NaCl) concentrations. As clear from the spectra the monolayer features are already formed at the 8mM SDS concentration indicating the surface is fully covered with $DS^-$ ions. This observation is fully in contrast with the SDS spectra at µM bulk concentration ranges. We don't observe any monolayer formation for pure SDS till 100 µM bulk concentration. Hence the addition of the NaCl doesn't have many repercussions in the HDVSFG spectra. We do see a steady decrease in the surface water signal as the direct consequence of the negative surface charge screening by the positive $Na^+$ ions.

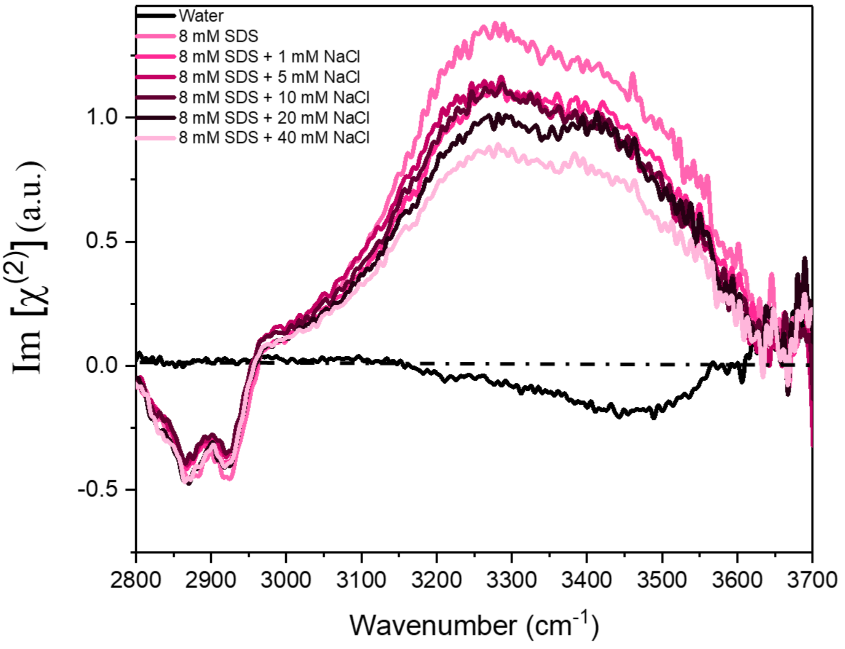


**Figure 2**: The HDVSFG spectra of SDS at CMC (8 mM bulk concentration) at various NaCl concentrations.

## SI 3: Modified Langmuir isotherm model used in this study

We calculated the adsorption of SDS to the interface with a modified Langmuir isotherm model. According to the standard Langmuir model, the interfacial occupancy of SDS molecules is given by: $\theta = \frac{K_{eq} * C_{sds}}{1 + K_{eq} * C_{sds}}$. where θ is the surface occupancy, $C_{sds}$ is the SDS bulk concentration, and $K_{eq}$ is the standard equilibrium constant of the adsorption process.

We modified this expression by introducing two additional terms, $K_{el}$ and $K_{vdW}$. The first term $K_{el}$ accounts for the energy associated with the nonzero surface potential, and the second term $K_{vdW}$ accounts for the van der Waals interactions between the aliphatic tails of the surfactants. The surface occupancy $\theta_{ds}$ of the $DS^-$ ions is thus given by:

$$\theta_{ds} = \frac{K_{eq} K_{el} K_{vdW} C_{sds}}{1 + K_{eq} K_{el} K_{vdW} C_{sds}} \quad (1)$$

$K_{el}$ depends on the surface potential:

$$K_{el} = e^{-\frac{e\phi}{k_b T}} \quad (2)$$

With $e$ the elementary electric charge, and $\phi$ the surface potential. The surface potential can be related to the surface charge density $\sigma$ using the Grahame equation. This equation follows from the Gouy-Chapman theory using the assumption that the total charge of the double layer equals

the negative charge of the surface charge. Using the one-dimensional Poisson equation and assuming that the gradient of the potential is equal to 0 at a large distances from the surface, the Grahame equation for single-valent ions can be derived:

$$\phi = \frac{2k_bT}{e}\sinh^{-1}\left(\frac{\sigma}{\sqrt{8C\varepsilon\varepsilon_0 k_bT}}\right) \tag{3}$$

Where $k_b$ is Boltzmann's constant, $\sigma$ is the surface charge density that depends on the surface occupancy $\theta_{ds}$ and the area $a$ occupied by each surfactant DS$^-$ ion ($\sigma D = e\theta_{ds}/a$), $C$ is the total salt concentration (SDS and NaCl) in mol/l, and $\varepsilon$ is the static permittivity of the solution. The hydrophobic interaction of the aliphatic tails is short-range and thus also strongly dependent on the surface occupancy $\theta_{ds}$. We modeled this interaction with the following expression:

$$K_{el} = e^{\frac{\Delta\, erf(5(\theta_{ds}-0.2)}{k_bT}}. \tag{4}$$

The exponential is a step function with $\theta_{ds}$= 0.2 as step value. The value of Δ was taken equal to $2k_bT$.

Equations (1) - (4) are coupled equations that are solved numerically for the surface occupancy $\theta_{ds}$ for a wide range of bulk SDS and NaCl concentrations using a Python script. "

**SI 4: HDVSFG spectra of 75 µM Dodecyltrimethylammonium bromide (DTAB) at different sodium chloride (NaCl) concentrations**.

This figure presents the HDVSFG spectra of the 75 µM DTAB at different NaCl concentrations. The stark change from the SDS spectra is due to the change in the surface charge (from negative to positive ) and that flips in the water dipole orientation at the interface. However, the trend in the water band located between 3200 and 3600 cm$^{-1}$ compared to pure DTAB remains the same as in the case of SDS.

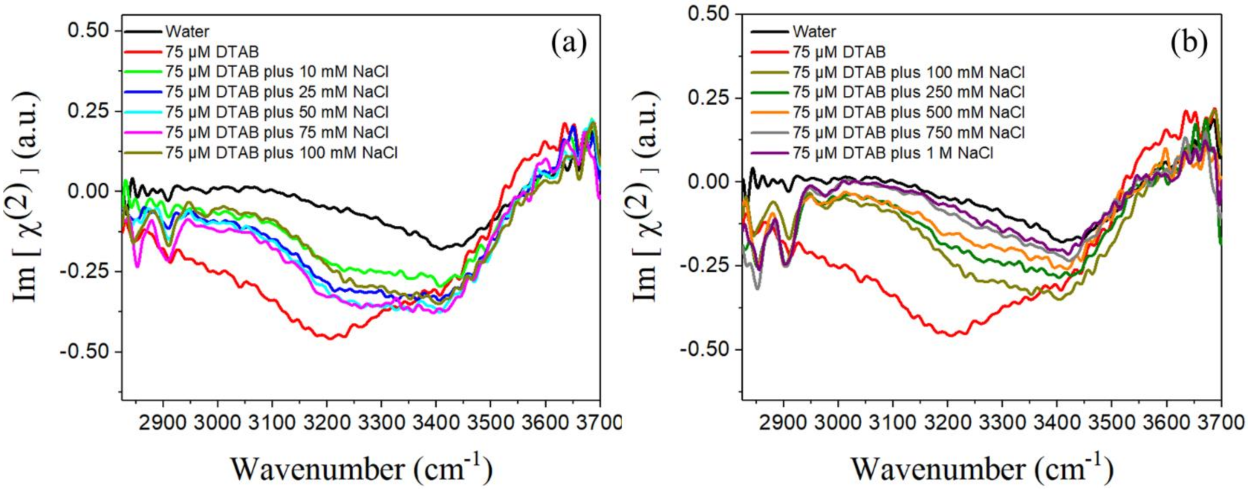


**Figure 3:** This figure shows the HDSFG response when different concentrations of NaCl were added to the 75 µM DTAB at the liquid/air interface. (a) shows the lower NaCl concentrations and (b) shows the higher NaCl concentrations.
