# Supplementary material for: Observation of a Two-Dimensional Hydrophobic Collapse at the Surface of Water Using Heterodyne-Detected Surface Sum-Frequency Generation
Source: J Phys Chem Lett. 2023 Oct 10;14(41):9285–90. doi: 10.1021/acs.jpclett.3c01530 (PMC10591499; doi:10.1021/acs.jpclett.3c01530)
Supplement: Supplementary file 2 — jz3c01530_si_003.pdf [file jz3c01530_si_003.pdf]

```
import csv

import symfit

import math

import numpy as np

import matplotlib.pyplot as plt

import scipy

import keyboard

import sympy

from numpy.fft import fft2, fftshift, ifftshift

from mpl_toolkits.axes_grid1 import make_axes_locatable

from matplotlib import animation

from scipy.optimize import curve_fit

from symfit import Parameter, parameters, variables, Model, Fit

from sympy import *

from sympy import Symbol

from sympy import nsolve

x = Symbol('x')


K0 = .08 #equilibrium constant SDS (uM-1)

K20 = 2000 #equilibrium constant c12e6 (mM-1)

num =40

cb = np.logspace(-6,0,num) #ionic strenght(M)

cb20 = 0*0.07 #c12e6 concentration (mM)

cs0 = np.linspace(25,100,num) #sds concentration (uM)

kbt = 25.7 #meV

RT = 2500 #J/mol

a = 1 #area per molecule (nm^^2)

x1 = [] # normalized SDS surface coverage

x2 = [] # normalized c12e6 surface coverage

sp = [] # surface potential

K0cc=[]
```

```
exp_c = [0.001,.02,.04,.06,.08,0.1,.25,.5,1]

exp_edl = [0.87,.8,.74,.7,.64,0.67,.49,.37,.24]

exp_c12e6 = [0.80,.6,.50,.45,.40,0.30,.25,.20,.15]


for cb0 in cb:

   # x1.clear()

   # x2.clear()

   # sp.clear()

   for cs in cs0:                                                                    #loop over sds concentration

       Kx = exp(-2*kbt*asinh(x*0.16/a/(sqrt(8000*RT*80*8.8*10**-12*(cb0+cs*10**-6))))/kbt)
#modulation of equilibrium constant SDS

       K0c = exp(2*(erf(5*(x-.2))+0)**1.0)

       equation = K0*K0c*Kx*cs/(1+K0*K0c*Kx*cs+K20*cb20) - x
#relation between surface coverage and surface potential

       solution = nsolve(equation,x,.4)                                        #solve numerial equation

       Kxs = exp(-2*kbt*asinh(float(solution)*0.16/a/(sqrt(8000*RT*80*8.8*10**-12*(cb0+cs*10**-
6))))/kbt)    #equilibrium constant SDS including surface potential

       K0cc.append(float(exp(2*(erf(5*(float(solution)-.2))+0)-0)))

       x2.append(K20*cb20/(1+K0*Kxs*cs+K20*cb20))                                #surface
coverage c12e6

       x1.append(float((solution)))                                            #surface coverage sds

       sp.append(float(2*kbt*asinh(float(solution)*0.16/a/(sqrt(8000*RT*80*8.8*10**-
12*(cb0+cs*10**-6))))))        #surface potential (mV)

testx1 = np.array(x1)

testx2 = np.array(x2)

testsp = np.array(sp)

testKc = np.array(K0cc)

test=testsp.reshape(num,num)

testx1=testx1.reshape(num,num)

testx2=testx2.reshape(num,num)

testKc=testKc.reshape(num,num)

test_t=test.transpose()
```

```
# plt.plot(cb,testx1[:,4])
plt.plot(cb,test[:,39])
plt.plot(cb,test[:,15])
plt.plot(cb,test[:,5])
plt.plot(cb,test[:,0])
# plt.plot(exp_c,np.array(exp_c12e6),'ro',label = 'IM_Chi2_EDL')
plt.ylim(0,130)
plt.xlabel('NaCl concentration (M)')
plt.ylabel('surface potential (mV)')
# plt.xscale('log')
plt.show()

# plt.plot(cb,testx1[:,4])
plt.plot(cb,testKc[:,39])
plt.plot(cb,testKc[:,15])
plt.plot(cb,testKc[:,5])
plt.plot(cb,testKc[:,0])
# plt.plot(exp_c,np.array(exp_c12e6),'ro',label = 'IM_Chi2_EDL')
# plt.ylim(0,100)
plt.xlabel('NaCl concentration (M)')
plt.ylabel('EC surface coverage (arb. u.)')
# plt.xscale('log')
plt.show()

# plt.plot(cb,testx1[:,4])
plt.plot(cb,test[:,39])
plt.plot(cb,test[:,15])
plt.plot(cb,test[:,6])
plt.plot(cb,test[:,0])
# plt.plot(exp_c,np.array(exp_c12e6),'ro',label = 'IM_Chi2_EDL')
```

```
# plt.ylim(0,100)
plt.xlim(-0.001,0.1)
plt.xlabel('NaCl concentration (M)')
plt.ylabel('surface potential (mV)')
# plt.xscale('log')
plt.show()

plt.plot(cb,testx1[:,29])
plt.plot(cb,testx1[:,13])
plt.plot(cb,testx1[:,5])
plt.plot(cb,testx1[:,0])
# plt.plot(cb,testx1)
# plt.plot(cb,testx2[:,4])
# plt.plot(exp_c,np.array(exp_c12e6),'ro',label = 'IM_Chi2_EDL')
plt.ylim(0,1)
plt.xlim(0,.1)
plt.xlabel('NaCl concentration (M)')
plt.ylabel('norm. surface coverage')
# plt.xscale('log')
plt.show()


plt.figure(1)
plt.imshow(testx1,extent=[10,100,0,-6],aspect='auto')
plt.title(label='')
plt.xlabel('SDS concentration (mM)')
plt.ylabel('ionic stengthe log(M)')
plt.colorbar(label='surface coverage (arb.u.)')
plt.show()

plt.figure(2)
```

```
plt.imshow(test,extent=[10,100,0,-6],aspect='auto')

plt.title(label='')

plt.xlabel('SDS concentration (mM)')

plt.ylabel('ionic stengthe log(M)')

plt.colorbar(label='surface potential (mV)')

plt.show()
```
