# Supplementary material for: Observation of a Two-Dimensional Hydrophobic Collapse at the Surface of Water Using Heterodyne-Detected Surface Sum-Frequency Generation
Source: J Phys Chem Lett. 2023 Oct 10;14(41):9285–90. doi: 10.1021/acs.jpclett.3c01530 (PMC10591499; doi:10.1021/acs.jpclett.3c01530)
Supplement: Supplementary file 3 — jz3c01530_si_004.pdf [file jz3c01530_si_004.pdf]

jz-2023-01530c.R1

Name: Peer Review Information for "Observation of a Two-Dimensional Hydrophobic Collapse at the Surface of Water using Heterodyne-Detected Surface Sum-Frequency Generation"

First Round of Reviewer Comments

Reviewer: 1

Comments to the Author

Sanghamitra Sengupta et al. present a HD-SFG study of the surfactant dodecyl sulfate at the interface with aqueous solutions with increasing NaCl concentration. The study aims at addressing the molecular details of surfact self assembly and the associated changes in the hydration water structure. The authors observe interesting changes in the HD-SFG spectra as a function of NaCl concentration in terms of both spectral shape (on both the CH and OH stretching regions) and amplitude. By focusing on the amplitude changes at a specific frequency (3250 cm-1), the authors find that the spectroscopic response of the interfacial water can be explained by a combination of hydrophobic tail-tail interactions that favour a dense surfactant packing (the “hydrophobic collapse” as in the title) at high concentration and the screening provided by Na+ cations in the liquid phase. The idea of a hydrophobic collapse is consistent with the spectral changes in the CH region.

My main concern with this overall potentially interesting manuscript is that some key spectroscopic changes are not addressed in the discussion and I fear that as a consequence the resulting interpretation could be an oversimplification of the problem, as detailed in the major and minor points below.

\- The authors discuss a non-linear dependence of the SFG intensity at 3250 cm-1 on the electrolyte concentration. They interpret it in terms of screening effect of the electrolyte, which in turn depends on the surface density of surfactants and on the electrolyte concentration. This interpretation is based on the fact that the SFG response of a charged interface can be expressed as the sum of a pure second-order term, which can be referred to as the chi2-BIL spectrum following the nomenclature introduced by Tian et al. in their seminal work on this topic [Phys. Rev. Lett. 2016, 116, 016101], plus an additional term due to the diffuse layer where "bulk-like" water is reoriented by the static field emanated by a charged surface (usually referred to as chi2-DL or chi3 contribution). All the discussions of the authors seem to be based on a simplified approach that only considers the chi2-DL term, while neglecting possible contributions from chi2-BIL. I understand that doing this allows a simpler qualitative interpretation of the experimental data. However, the only case where such an approach is qualitatively correct is when the spectroscopic changes in the BIL are negligible compared to that in the DL, i.e. where the potential difference across the DL is large enough and interferences are small. If it is the case, the HD-SFG spectrum must look like the well-known chi3 spectrum of water, with the typical 2-band structure in the OH-stretching region with maxima at ~3200 and ~3400 cm-1. See e.g. refs.[Phys. Rev. Lett. 2016, 116, 016101, J. Phys. Chem. Lett. 2023, 14, 1301, https://doi.org/10.1021/acs.jpclett.2c03300] for details and some examples. This is clearly not the case for the spectra presented in this manuscript. Therefore, the authors must be more careful in their analysis. I advise them to correctly separate the BIL and DL contributions (e.g. with one of the experimental approaches available

nowadays, e.g.[Phys. Rev. Lett. 2016, 116, 016101; J. Phys. Chem. Lett. 2018, 9, 4109, 10.1021/acs.jpclett.8b01650], or with the help of theory, e.g. [https://doi.org/10.1021/acs.jpclett.2c03300, 10.1039/C7CP01978E, DOI https://doi.org/10.1039/C7CP06110B]). Without doing this, their conclusions remain speculative, although reasonable.

\- Why did the authors select the 3250 cm-1 frequency for their analysis? What happens with a different frequency? Again, any frequency would be good in cases where chi2-BIL is negligible, as the HD-SFG spectrum would be essentially given by the chi3 spectrum of bulk water scaled by a complex surface potential term [Phys. Rev. Lett. 2016, 116, 016101, Nat. Commun. 2016, 7, 13587, J. Phys. Chem. C 2016, 120, 9165−9173,]. However, looking at the present spectra it seems not to be the case here.

\- More generally, the authors ignore the remarkable changes in the shape of their ImChi2 spectra with increasing [NaCl]. These are likely arising From the compensation of BIL and DL contributions, as well as changes in the BIL water structure in direct contact with the surfactant layer. Understanding such interplay of BIL/DL spectra is crucial for a correct interpretation (see e.g. [Phys. Rev. Lett. 2016, 116, 016101; https://doi.org/10.1021/acs.jpclett.2c03300; 10.1021/acs.jpclett.8b01650]) I find this is the most lacking aspect of the present manuscript.

As a minor point, when discussing the CH contribution, the authors connect the HD-SFG intensity directly to surface density of surfactants. However, also the orientation of the hydrophobic tail will have a huge impact on the intensity, possibly causing large changes without any

increase in surface density, see e.g. [J. Chem. Phys. 2019, 150, 204708]. Could you elaborate on this?

As a curiosity, would it be possible to show the spectra until 3750 cm-1?

The authors discuss the hydrophobic collapse in terms of vdw interactions between hydrophobic tails. This is correct if the tails stick totally out of the liquid phase. If not, even if the tails are only partially hydrated, the free energy will be dominated by the dewetting of the tails (i.e. by the cavitation free energy) more than by vdw interactions. As a rule of thumb, if the tails are at least partially hydrated one would expect a positive ImChi2 band at ~3650, but unfortunately this cannot be confirmed because the spectra are not shown in the >3600 frequency range. If it is the case, the discussion and physical model could be adapted to take cavitation free energy into account.

In summary, I think this is a potentially interesting work, but the above mentioned concerns should be addressed before it can be considered for publication in JPCL, we well as in any other journal.

Reviewer: 2

Comments to the Author

Sengupta et al studied the salt effect on the air/SDS/water interface with the SDS concentration much lower CMC using HD-VSFG spectroscopy. With a low SDS concentration of <1mM, the amplitude of the positive OH stretch band exhibits a peculiar salt concentration dependence: A drastic rapid decrease, recovery, and then gradual decrease. They rationalize this interesting salt-dependent spectral change in terms of two types of the counter ion (Na+) effect, i.e., the vertical electric field screening to suppress

the diffused layer and the horizontal screening that allows more concentration of SCS at the water surface. The amplitude of the CH stretch, which is expected to reflect the SDS concentration at the surface, supports the latter screening effect. They also carried out model calculations based on thermodynamics, which looks consistent with their interpretation.

The experimental observation is interesting and the main arguments of the authors sound reasonable. However, as I write below, this paper has numbers of problems, including insufficient explanation. From the viewpoint of the topic, I do not think that it needs urgency and that it is more suitable for a full paper. Therefore, I suggest submitting this paper to JPC A/B/C after making relevant revisions for the points I list below.

1.

The authors should perform surface tension measurements, which provide information about the surface excess. They discuss the SDS concentration at the surface based on the amplitude of the CH stretch but it reflects not only concentration but also the degree of the orientation. The separation of these two factors is crucial in particular for the very low SDS concentration that is adopted in this study.

2.

The authors need to clearly write the phase of the SDS monolayer in this study. I suppose it is the gas phase.

3.

With no or very low salt concentration, the OH stretch band of the Im chi(2) spectra is distorted due to the contribution of the oriented water in a deep region. Therefore, the authors need to “correct” the spectral distortion to evaluate the amplitude of the OH stretch band as they properly mention in the paper. However, they do not describe how they did at all and do not show the spectra after correction. The relevant information is critical because the salt-dependent amplitude change in question appears in the low salt concentration region.

4.

Relating to #3, their Im chi(2) spectrum without salt is very different from the air/SDS/water interface above CMC. Because the Im chi(2) spectra above or near CMC have been reported in many papers, the authors should cite them and mention that difference arises from the low SDS concentration they adopted. It is important to avoid unnecessary confusion.

5.

In the 2nd paragraph on page 5, they write, “The added Na+ … (Z direction).” This description contradicts the picture of the diffused layer (Gouy-Chapman layer) in which Na+ is diffused in the quite deep region in the water phase. Do the authors disagree with the GC layer picture and wish to claim that only the Stern layer is formed at the SDS/water interface under their condition? Clarification of the authors' argument is necessary.

6.

The explanation of their simulation (Figure 4) given in SI is very insufficient. It is very difficult to follow. A much more detailed description is necessary.

7.

I strongly believe that the authors should not use the term “hydrophobic collapse” in this paper. It is because hydrophobic collapse is an established term that is used for proteins to form tertiary structures with hydrophobic cores by interaction with water.

8.

The authors cite ref 24 and 25 as references for HD-VSFG but it is inadequate. In fact, the authors’ measurements were not performed using the method reported in ref 24, and ref 25 is the paper of the authors’ own group. More appropriate references, probably review articles, should be cited here, e.g., 1) Shen, Y. R., Phase-Sensitive Sum-Frequency Spectroscopy. Annu. Rev. Phys. Chem. 2013, 64, 129-150. 2) Nihonyanagi, S.; Mondal, J. A.; Yamaguchi, S.; Tahara, T., Structure and Dynamics of Interfacial Water Studied by Heterodyne-Detected Vibrational Sum-Frequency Generation. Annu. Rev. Phys. Chem. 2013, 64, 579-603.

9.

Ref. 26 and 27 are missing in the main text.

Author's Response to Peer Review Comments:

Observation of a Two-Dimensional Hydrophobic Collapse at the Surface of Water using Heterodyne-Detected Surface Sum-Frequency Generation

Reviewers’ comments reply

<u>Reviewer 1</u>

We thank the reviewer for carefully reading our manuscript and for his/her/their useful comments and suggestions. Below we will address the points raised by the reviewer in the order in which they appeared in the report.

<u>Reviewer comment</u>

The authors discuss a non-linear dependence of the SFG intensity at 3250 cm-1 on the electrolyte concentration. They interpret it in terms of screening effect of the electrolyte, which in turn depends on the surface density of surfactants and on the electrolyte concentration. This interpretation is based on the fact that the SFG response of a charged interface can be expressed as the sum of a pure second-order term, which can be referred to as the chi2-BIL spectrum following the nomenclature introduced by Tian et al. in their seminal work on this topic [Phys. Rev. Lett. 2016, 116, 016101], plus an additional term due to the diffuse layer where "bulk-like" water is reoriented by the static field emanated by a charged surface (usually referred to as chi2-DL or chi3 contribution). All the discussions of the authors seem to be based on a simplified approach that only considers the chi2-DL term, while neglecting possible contributions from chi2-BIL. I understand that doing this allows a simpler qualitative interpretation of the experimental data. However, the only case where such an approach is qualitatively correct is when the spectroscopic changes in the BIL are negligible compared to that in the DL, i.e. where the potential difference across the DL is large enough and interferences are small. If it is the case, the HD-SFG spectrum must look like the well-known chi3 spectrum of water, with the typical 2-band structure in the OH-stretching region with maxima at ~3200 and ~3400 cm-1. See e.g. refs.[Phys. Rev. Lett. 2016, 116, 016101, J. Phys. Chem. Lett. 2023, 14, 1301, https://eur03.safelinks.protection.outlook.com/?url=https%3A%2F%2Fdoi.org%2F10.1021%2Facs.jpclett.2c03300&data=05%7C01%7C%7C3634b5cfaf0b41f2099c08db86cfae58%7Cd1598a4048ac4fedb8b93dcb440ac6fa%7C0%7C0%7C638251999885066434%7CUnknown%7CTWFpbGZsb3d8eyJWIjoiMC4wLjAwMDAiLCJQIjoiV2luMzIiLCJBTiI6Ik1haWwiLCJXVCI6Mn0%3D%7C3000%7C%7C%7C&sdata=dAAIG7fC5xfE%2BRofs5Dw8W2bF%2FskaRxdFl5SS3neYOM%3D&reserved=0] for details and some examples. This is clearly not the case for the spectra presented in this manuscript. Therefore, the authors must be more careful in their analysis. I advise them to correctly separate the BIL and DL contributions (e.g. with one of the experimental approaches available nowadays, e.g.[Phys. Rev. Lett. 2016, 116, 016101; J. Phys. Chem. Lett. 2018, 9, 4109, 10.1021/acs.jpclett.8b01650], or with the help of theory, e.g. [https://eur03.safelinks.protection.outlook.com/?url=https%3A%2F%2Fdoi.org%2F10.1021%2Facs.jpclett.2c03300&data=05%7C01%7C%7C3634b5cfaf0b41f2099c08db86cfae58%7Cd1598a4048ac4fedb8b93dcb440ac6fa%7C0%7C0%7C638251999885066434%7CUnknown%7CTWFpbGZsb3d8eyJWIjoiMC4wLjAwMDAiLCJQIjoiV2luMzIiLCJBTiI6Ik1haWwiLCJXVCI6Mn0%3D%7C3000%7C%7C%7C&sdata=dAAIG7fC5xfE%2BRofs5Dw8W2bF%2FskaRxdFl5SS3neYOM%3D&reserved=0, 10.1039/C7CP01978E,

DOI https://eur03.safelinks.protection.outlook.com/?url=https%3A%2F%2Fdoi.org%2F10.1039%2FC7CP06110B&data=05%7C01%7C%7C3634b5cfaf0b41f2099c08db86cfae58%7Cd1598a4048ac4fedb8b93dcb440ac6fa%7C0%7C0%7C638251999885066434%7CUnknown%7CTWFpbGZsb3d8eyJWIjoiMC4wLjAwMDAiLCJQIjoiV2luMzIiLCJBTiI6Ik1haWwiLCJXVCI6Mn0%3D%7C3000%7C%7C%7C&sdata=syIst4SwV8VWX4G0exAifAvOBpDZYiDKR%2BZQMjS%2B9Ms%3D&reserved=0]). Without doing this, their conclusions remain speculative, although reasonable.

Authors reply

We thank the reviewer for making this interesting and important comment. In the paper by Y.C. Wen et al. (Phys. Rev. Lett. **116**, 016101 (2016)) a method is presented to distinguish the sum-frequency generation response of the bonded interface layer (BIL) from that of the diffuse layer (DL). In that work the surface charge density was varied by varying the pH of the solution, thereby changing the surfactant from a neutral state to a negatively charged state. In our work, we are studying the effect of salt on the response of a surface that is (partly) covered with a surfactant that is always negatively charged. Concerning the surface charge density, our work thus corresponds to the case of the paper of Wen et al. at high pH. Following this approach, we find that for NaCl concentrations up to a few hundred millimolar, the sum-frequency generation response is completely dominated by the DL response. This is evident from the fact that up to a NaCl concentration of 250 mM, the shape of the spectrum does not change.

At concentrations >500 mM the spectrum shows a slight distortion, in particular a shift to higher frequencies, and this could indeed be due to the relative increase of the contribution of the BIL response, as suggested by the reviewer. The BIL response represents the response of water molecules hydrogen-bonded to the sulfate groups of the DS^- surfactant and these will have a response at slightly higher frequencies than hydrogen-bonded bulk water molecules. Regarding the present work, it is important to note that the observed strongly nonlinear dependence on the salt concentration solely concerns the DL response, as this nonlinearity is observed in the concentration range up to 100 mM, where the signal is dominated by this response.

Action taken

In the revised manuscript we included references to Y-C. Wen et al. (Phys. Rev. Lett. **116**, 016101 (2016)), and S. Pezotti et al. (Physical Chemistry Chemical Physics **20**, 5190-5199 (2018)) describing the BIL and DL responses. We also included the following texts on pages 4 and 5 of the revised manuscript:

'For concentrations up to a NaCl concentration of 250 mM, the shape of the O-H stretch spectrum does not change, and only the amplitude changes. For concentrations >500 mM the spectrum shows a slight distortion, in particular a shift to higher frequencies. In interpreting this spectral change we follow the work of Refs. 27-29. In these works, it was demonstrated that the spectrum of the O-H vibrations of water underneath a (charged) surfactant layer is formed by the sum of the contribution of a bonded interface layer (BIL), representing water molecules that are

directly bonded to the interface and the contribution of water molecules in the diffuse layer (DL) below the interface. At low salt concentrations (<250 mM) we expect the response of the DL layer to dominate, as is corroborated by the fact that the spectral shape does not change in this concentration range. We find that in this region the $\mathrm{Im}(\chi^{(2)})$ spectrum of water is largely formed by a broad response centered at 3250 cm^{-1}. The change of the spectral shape observed for NaCl concentrations >250 mM is likely due to the increased relative contribution of the BIL response, due to the fact that the DL response will become small at these higher concentrations due to screening of the surface electric field. The BIL response will be dominantly formed by water molecules hydrogen-bonded to the sulfate groups of the DS^- surfactant. These hydrogen bonds will likely be somewhat weaker than those between water molecules in the bulk, thus explaining the small blue shift of the spectrum.', and on page 5 of the revised manuscript:

'It should be noted that in the salt concentration regions (i) and (ii) the water OH stretch signal is completely dominated by the DL response, which implies that the observed nonlinear dependence of the amplitude of the signal on the salt concentration is not the result of a change of the contribution of the BIL response to the signal.'.

Reviewer comment

Why did the authors select the 3250 cm-1 frequency for their analysis? What happens with a different frequency? Again, any frequency would be good in cases where chi2-BIL is negligible, as the HD-SFG spectrum would be essentially given by the chi3 spectrum of bulk water scaled by a complex surface potential term [Phys. Rev. Lett. 2016, 116, 016101, Nat. Commun. 2016, 7, 13587, J. Phys. Chem. C 2016, 120, 9165−9173,]. However, looking at the present spectra it seems not to be the case here.

Authors Reply

The BIL response is negligible in the concentration range up to 250 mM, as is clear from the fact that the shape of the OH stretch spectrum shows negligible changes in this concentration range, as indeed is to be expected for a DL-dominated response. Hence, we could indeed have chosen another frequency to illustrate the strongly nonlinear dependence of the water signal on the salt concentration in the concentration range up to 100 mM.

Reviewer comment:

More generally, the authors ignore the remarkable changes in the shape of their ImChi2 spectra with increasing [NaCl]. These are likely arising From the compensation of BIL and DL contributions, as well as changes in the BIL water structure in direct contact with the surfactant layer. Understanding such interplay of BIL/DL spectra is crucial for a correct interpretation (see e.g. [Phys. Rev. Lett. 2016, 116, 016101;

https://eur03.safelinks.protection.outlook.com/?url=https%3A%2F%2Fdoi.org%2F10.1021%2Facs.jpclett.2c03300&data=05%7C01%7C%7C3634b5cfaf0b41f2099c08db86cfae58%7Cd1598a4048ac4fedb8b93dcb440ac6fa%7C0%7C0%7C638251999885066434%7CUnknown%7CTWFpbGZsb3d8eyJWIjoiMC4wLjAwMDAiLCJQIjoiV2luMzIiLCJBTiI6Ik1haWwiLCJXVCI6Mn0%3D%7C3000%7C%7C%7C&sdata=dAAIG7fC5xfE%2BRofs5Dw8W2bF%2FskaRxdFl5SS3neYOM%3D&reserved=0; 10.1021/acs.jpclett.8b01650]) I find this is the most lacking aspect of the present manuscript.

Authors Reply

We would like to thank the reviewer again for pointing out that the change of the Im(□$^{(2)}$) spectrum at high salt concentrations may be due to an increase of the relative contribution of the BIL response. We included this explanation in the revised manuscript, also including an explanation for the small difference between the DL and the BIL responses. As argued in our reply to the first point, it is important to note that the strongly nonlinear dependence of the water signal on the salt concentration observed in the concentration range up to 100 mM, cannot be explained from a competition between BIL and DL responses. This nonlinear dependence results from the effects of the added ions on the in-plane Coulomb repulsion and the van der Waals interaction of the DS^- surfactants that in turn strongly affect the response of the CH vibrations and the DL water response.

Reviewer comment

As a minor point, when discussing the CH contribution, the authors connect the HD-SFG intensity directly to surface density of surfactants. However, also the orientation of the hydrophobic tail will have a huge impact on the intensity, possibly causing large changes without any increase in surface density, see e.g. [J. Chem. Phys. 2019, 150, 204708]. Could you elaborate on this?

Authors reply

We thank the reviewer for this comment and for pointing out the J. Chem. Phys. Article to us. We agree that the HDVSFG signal of the CH vibrations is determined both by the surfactant surface density and by the average orientation of the hydrophobic tails of the surfactants. The enhanced surfactant surface density will likely induce the surfactants to get better packed and their hydrophobic tails to get more oriented perpendicular to the surface, which will enhance the CH signals.

Action taken

We added a reference to the J. Chem. Phys paper cited by the reviewer. We also added the following sentence to page 6 of the revised manuscript:
'In addition to the increase in surface density, the amplitude of the C-H stretching bands likely also increases because of an enhanced orientation of the aliphatic tails of the DS^- ions perpendicular to the surface[32].'.

Reviewer comment

As a curiosity, would it be possible to show the spectra until 3750 cm-1?

Authors reply
It is not possible with our setup to measure high-quality spectra between 2800 and 3750 cm^{-1} in one go. As the quantitative data analysis requires a good signal-to-noise in the C-H stretching region, we could thus not extend the frequency axis to 3750 cm^{-1}.

Reviewer comment
The authors discuss the hydrophobic collapse in terms of vdw interactions between hydrophobic tails. This is correct if the tails stick totally out of the liquid phase. If not, even if the tails are only partially hydrated, the free energy will be dominated by the dewetting of the tails (i.e. by the cavitation free energy) more than by vdw interactions. As a rule of thumb, if the tails are at least partially hydrated one would expect a positive ImChi2 band at ~3650, but unfortunately this cannot be confirmed because the spectra are not shown in the >3600 frequency range. If it is the case, the discussion and physical model could be adapted to take cavitation free energy into account.

Authors reply
Following the reasoning of the reviewer there could be water molecules hydrating the tails at low salt concentrations that would vanish at high salt concentrations when the tails become dewetted. The response of water molecules hydrating the tails would be at relatively high frequencies (as these involve weak hydrogen bonds). However, we would expect this band to be a negative Im(□$^{(2)}$) band, as these water molecules will have a net orientation with their OH groups pointing down to the negative sulfate head group of the DS^- surfactant. We do not observe such a negative band at high frequencies at low salt concentrations. It could be that the weak negative Im(□$^{(2)}$) band is concealed by the broad positive response of water molecules that are below the sulfate groups, so we can neither rule out nor confirm the suggestion of the reviewer.

Reviewer comment
In summary, I think this is a potentially interesting work, but the above mentioned concerns should be addressed before it can be considered for publication in JPCL, we well as in any other journal.

Authors reply
We thank the reviewer for his/her/their expert review and highly useful comments and suggestions. We hope that we have addressed all the points of the report adequately in the revised version of our manuscript.

Reviewer 2
We thank the reviewer for carefully reading our manuscript and for his/her/their useful comments and suggestions. Below we will address the points raised by the reviewer in the order in which they appeared in the report.

Reviewer comment

The authors should perform surface tension measurements, which provide information about the surface excess. They discuss the SDS concentration at the surface based on the amplitude of the CH stretch but it reflects not only concentration but also the degree of the orientation. The separation of these two factors is crucial in particular for the very low SDS concentration that is adopted in this study.

Authors reply

We agree with the reviewer that the CH signals depend both on the surfactant surface density and on the degree of orientation of the surfactant hydrophobic tails. We added this notion to the revised manuscript. We also agree with the reviewer that the surface tension is indeed often an excellent measure of the surface density. However, in our work, we are dealing with an ionic surfactant and with the effects of the addition of a significant concentration of other ions that also affect the surface tension. The observed surface tension is thus a combined effect of the surfactants, the counter ions of the surfactants, and the added Na^+ and Cl^- ions, which makes it very difficult to relate the surface tension to the DS^- surfactant surface density, and to determine changes in this density upon adding NaCl. Therefore, for this system, the measurement of the response of the CH stretch vibrations is a more useful measure for the surface density, albeit that this response is indeed also influenced by the orientation of the aliphatic groups of the surfactant.

Action taken

We also added the following sentence to page 6 of the revised manuscript:
'In addition to the increase in surface density, the amplitude of the C-H stretching bands likely also increases because of an enhanced orientation of the aliphatic tails of the DS^- ions perpendicular to the water/air interface[32].'.

Reviewer comment

The authors need to clearly write the phase of the SDS monolayer in this study. I suppose it is the gas phase.

Authors reply

All measurements were done at the liquid/air interface.

Action taken

We included the following text on page 3 of the revised manuscript: 'In this work, we examine the interaction between SDS and NaCl at different SDS and NaCl bulk concentrations using heterodyne detected vibrational sum frequency generation spectroscopy[23-24] (HD-VSFG) at the liquid-air interface'.

Reviewer comment

With no or very low salt concentration, the OH stretch band of the Im chi(2) spectra is distorted due to the contribution of the oriented water in a deep region. Therefore, the authors need to "correct" the spectral distortion to evaluate the amplitude of the OH stretch band as they properly mention in the paper. However, they do not describe how they did at all and do not show

the spectra after correction. The relevant information is critical because the salt-dependent amplitude change in question appears in the low salt concentration region.

Authors Reply

The Im(□$^{(2)}$) spectrum of SDS in water is indeed distorted if there is no further added salt, as shown in Figure 1a. This phase distortion vanishes already when adding 2 mM of NaCl due to the drastic decrease of the Debye screening length. Hence, there was no need to correct the spectra for this effect, we merely show the distorted spectrum for the case of no added salt, to illustrate the effect.

Reviewer comment

Relating to #3, their Im chi(2) spectrum without salt is very different from the air/SDS/water interface above CMC. Because the Im chi(2) spectra above or near CMC have been reported in many papers, the authors should cite them and mention that difference arises from the low SDS concentration they adopted. It is important to avoid unnecessary confusion.

Authors reply

We thank the reviewer for this suggestion. Indeed the Im(□$^{(2)}$) spectra measured for SDS concentrations near or above CMC (8 mM) differ from the spectra measured in the µM range. This is because at mM concentrations of SDS, the screening becomes sufficiently strong, and the Debye screening length is thus sufficiently short, to have no phase distortion.

Action taken

We added the following sentence to the revised manuscript at the bottom of page 4 after we introduced the HDVSFG spectra of the lower SDS concentration:
'This phase distortion is not present in the HDVSFG spectrum of a solution of SDS at its critical micelle concentration (CMC) of 8 mM', as was also found in Ref. 26, because then the Debye screening length becomes sufficiently short (see also figure SI 2).'.
We also added the figure below to the supplementary information of the manuscript (Figure SI 3).

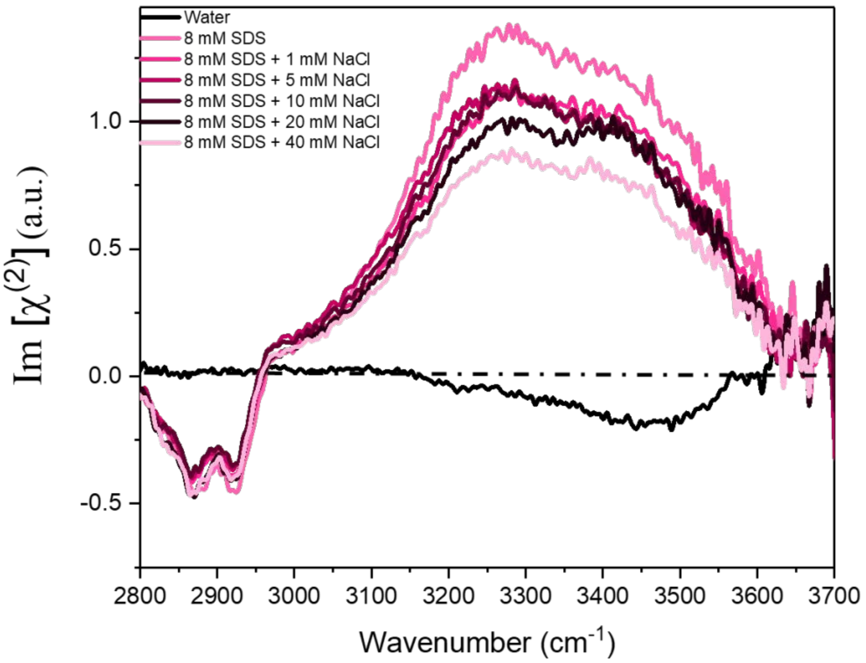

Figure SI2: The HDVSFG spectra of a solution of 8 mM SDS and different NaCl concentrations.

Reviewer comment

In the 2nd paragraph on page 5, they write, "The added Na+ ... (Z direction)." This description contradicts the picture of the diffused layer (Gouy-Chapman layer) in which Na+ is diffused in the quite deep region in the water phase. Do the authors disagree with the GC layer picture and wish to claim that only the Stern layer is formed at the SDS/water interface under their condition? Clarification of the authors' argument is necessary.

Authors reply

We thank the reviewer for making this comment. Indeed the wording in the previous version of the manuscript was not correct. The reviewer is correct in pointing out that the Na^+ ions diffuse quite deep in the water phase. This is in fact exactly how we modeled the observed effects of adding NaCl to the solution, i.e. by using the Grahame equation, consistent with the GouyChapman picture of the interface.

Action taken

We corrected the text in the page number 6 in the revised manuscript to: 'The added Na^+ ions will have a screening effect and decrease the length over which this field penetrates the bulk (in the Z direction).'.

Reviewer comment

The explanation of their simulation (Figure 4) given in SI is very insufficient. It is very difficult to follow. A much more detailed description is necessary.

Authors reply

We agree with the reviewer that the description of the theoretical model in the SI was incomplete. We corrected this in the SI of the revised manuscript.

Action taken

We added the following text to the SI:

"**Modified Langmuir isotherm model**

We calculated the adsorption of SDS to the interface with a modified Langmuir isotherm model. According to the standard Langmuir model, the interfacial occupancy of SDS molecules is given by: $\theta = \frac{K_{eq}*C_{sds}}{1 + K_{eq}*C_{sds}}$. where θ is the surface occupancy, C_{sds} is the SDS bulk concentration, and K_{eq} is the standard equilibrium constant of the adsorption process.

We modified this expression by introducing two additional terms, K_{el} and K_{vdW}. The first term K_{el} accounts for the energy associated with the nonzero surface potential, and the second term K_{vdW} accounts for the van der Waals interactions between the aliphatic tails of the surfactants. The surface occupancy θ_{ds} of the DS^- ions is thus given by:

$$\theta_{ds} = \frac{K_{eq}K_{el}K_{vdW}C_{sds}}{1 + K_{eq}K_{el}K_{vdW}C_{sds}} \quad (1)$$

K_{el} depends on the surface potential:

$$K_{el} = e^{-\frac{e\phi}{k_bT}} \quad (2)$$

With the elementary electric charge, and e ϕ the surface potential. The surface potential can be related to the surface charge density using the Grahame equation. This equation follows σ from the Gouy-Chapman theory using the assumption that the total charge of the double layer equals the negative charge of the surface charge. Using the one-dimensional Poisson equation and assuming that the gradient of the potential is equal to 0 at a large distances from the surface, the Grahame equation for single-valent ions can be derived:

$$\phi = \frac{2k_bT}{e}\sinh^{-1}\left(\frac{\sigma}{\sqrt{8C\varepsilon\varepsilon_0k_bT}}\right) \quad (3)$$

Where k_b is Boltzmann's constant,   is the surface charge density that depends on the surface σ occupancy θ_{ds} and the area a occupied by each surfactant DS^- ion ($\sigma D = e\theta_{ds}/a$), C is the total salt concentration (SDS and NaCl) in mol/l, and is the static permittivity of the ε solution. The hydrophobic interaction of the aliphatic tails is short-range and thus also strongly dependent on the surface occupancy θ_{ds}. We modeled this interaction with the following expression:

$$K_{el} = e^{\frac{\Delta\, erf(5(\theta_{ds} - 0.2)}{kbT}}. \qquad (4)$$

The exponential is a step function with θ_{ds}= 0.2 as step value. The value of Δ was taken equal to $2k_bT$.

Equations (1) - (4) are coupled equations that are solved numerically for the surface occupancy θ_{ds} for a wide range of bulk SDS and NaCl concentrations using a Python script. "

Reviewer comment

I strongly believe that the authors should not use the term "hydrophobic collapse" in this paper. It is because hydrophobic collapse is an established term that is used for proteins to form tertiary structures with hydrophobic cores by interaction with water.

Authors reply

We agree with the reviewer that the term "hydrophobic collapse" is usually used in a different context (e.g. protein folding), and is largely driven by favorable interactions between water molecules. However, also in that context favorable van der Waals interactions of the hydrophobic groups usually contribute to the effect. In our study the strong increase in surface density results from the in-plane screening of the Coulomb repulsion of the headgroups, leading to the enabling of the favorable short-range van der Waals interaction of the hydrophobic tails of the surfactants. This increase in surface density can very well be regarded as a collapse of the surfactants on each other in the plane of the surface. Therefore we would like to keep the elements "hydrophobic" and "collapse", but also emphasize the fact that this happens in a (surface) plane, hence the term "2D hydrophobic collapse".

Reviewer comment

The authors cite ref 24 and 25 as references for HD-VSFG but it is inadequate. In fact, the authors' measurements were not performed using the method reported in ref 24, and ref 25 is the paper of the authors' own group. More appropriate references, probably review articles, should be cited here, e.g., 1) Shen, Y. R., Phase-Sensitive Sum-Frequency Spectroscopy. Annu. Rev. Phys. Chem. 2013, 64, 129-150. 2) Nihonyanagi, S.; Mondal, J. A.; Yamaguchi, S.; Tahara, T., Structure and Dynamics of Interfacial Water Studied by Heterodyne-Detected Vibrational Sum-Frequency Generation. Annu. Rev. Phys. Chem. 2013, 64, 579-603.

Authors reply

We thank the reviewer for pointing out this error.

Action taken
We removed references 24 and 25 and included the references suggested by the reviewer (references 23 and 24 in the revised manuscript).

Reviewer comment: *Ref. 26 and 27 are missing in the main text.*

Authors Reply:
We thank the reviewer for pointing this out. We have carefully checked all the references and corrected the errors

jz-2023-01530c.R2

Name: Peer Review Information for "Observation of a Two-Dimensional Hydrophobic Collapse at the Surface of Water using Heterodyne-Detected Surface Sum-Frequency Generation"

Second Round of Reviewer Comments

Reviewer: 1

Comments to the Author

The authors made efforts in addressing most of the reviewer comments, and overall the manuscript is improved in my opinion. However, I'm still not fully convinced by the authors interpretation that the signal below 100 mM NaCl is dominated by the DL/chi3 contribution. Although very reasonable, this picture do not fit completely with their own spectra in my opinion. In particular, I still have two concerns that remain to be addressed before I can recommend publication

1. The shape of the Im chi3 spectrum is known, and it never changes sign in the OH stretching frequency range. However, in the present experiments, already with 20 and 25 mM, ImChi2 changes sign (from positive to negative) at above 3300 cm-1. This is not consistent with the idea of a chi3 dominated spectrum. Could the authors elaborate on this?

2. There is a dramatic change in the shape between no salt and + 10 mM NaCl. How do the authors rationalize this? It does not appear as a standard change in the DL-contribution, which as stated by the authors would appear in an intenistiy modulation but no change in the spectral shape. It does not seem to be something that can be explained by interferences either, since the Im Chi2 intensity decreases instead of increasing by adding 10 mM NaCl.

Reviewer: 2

Comments to the Author

Sengupta et al made revisions according to the comments of this reviewer, and the paper is now much more readable. Most of their responses are satisfactory, but they completely misunderstood one of my comments.

In my second comment, I encouraged them to “clearly write the phase of the SDS monolayer in this study.” In a surface tension–area isotherm of a monolayer, as the area per molecule is reduced, the monolayer adopts gas, liquid-expanded (LE), liquid-condensed (LC), condensed/solid (C) phases before collapsing from the interface. I supposed that the monolayer examined in this study was the gas phase in this sense.

The authors need to provide relevant discussion on the phase in the above-mentioned meaning.

Author's Response to Peer Review Comments:

Observation of a Two-Dimensional Hydrophobic Collapse at the Surface of Water using Heterodyne-Detected Surface Sum-Frequency Generation

Reviewers’ comments reply

Reviewer 1

We thank the reviewer for again carefully reading our manuscript and for his/her/their useful comments and suggestions. Below we will address the points raised by the reviewer in the order in which they appeared in the report.

Reviewer comment

The authors made efforts in addressing most of the reviewer comments, and overall the manuscript is improved in my opinion. However, I'm still not fully convinced by the authors interpretation that the signal below 100 mM NaCl is dominated by the DL/chi3 contribution. Although very reasonable, this picture do not fit completely with their own spectra in my opinion. In particular, I still have two concerns that remain to be addressed before I can recommend publication

1. The shape of the Im chi3 spectrum is known, and it never changes sign in the OH stretching frequency range. However, in the present experiments, already with 20 and 25 mM, ImChi2 changes sign (from positive to negative) at above 3300 cm-1. This is not consistent with the idea of a chi3 dominated spectrum. Could the authors elaborate on this?

Author reply

The negative sign of the spectrum at frequencies >3300 cm^{-1} of a solution containing 25 μM of SDS and very little NaCl is partly the result of the fact that the surface density of DS^- is very low, and partly due to phase distortion. At this SDS concentration (and little NaCl added) the surface electric field exerted by the surfactant ions is weak and thus the Im($\chi^{(2)}$) spectrum will still show similarity with the Im($\chi^{(2)}$) spectrum of pure H_2O that is negative up to frequencies of 3550 cm^{-1} .(see for instance Shen, Y. R., Phase-Sensitive Sum-Frequency Spectroscopy. *Annu. Rev. Phys. Chem.* **2013,** *64* (1), 129-150). In addition, for a solution containing 25 μM SDS and up to a few mM of NaCl, the Debye screening length is long and the SFG signal originates to a large part from regions deeper down in the solution, leading to a phase distortion. Upon adding NaCl, the surface density of DS^- gets higher, and the electric field exerted by the surfactant ions gets stronger. At the same time, the Debye length becomes shorter so that the phase distortion vanishes. As a result, at NaCl concentrations > 25 mM, the spectrum acquires the shape of the Im($\chi^{(3)}$) spectrum of the diffuse layer (DL). At NaCl concentrations >500 mM the spectrum again shows a slight distortion, in particular a shift to higher frequencies, which is likely due to the relative increase of the contribution of the BIL response, as was suggested by the reviewer, and for which we are grateful to the reviewer.

Action taken

In the revised manuscript we extended the description of the Im($\chi^{(2)}$) spectrum observed at low NaCl concentrations:

"For a solution containing 25 μM SDS and a low concentration of NaCl, the Im($\chi^{(2)}$) spectrum has a negative sign at frequencies >3300 cm^{-1}. For these solutions, the surface density of DS^- ions is low and the surface electric field exerted by these ions is weak. The Im($\chi^{(2)}$) spectrum will thus show similarity with the Im($\chi^{(2)}$) spectrum of pure H_2O that is negative in the OH stretch vibrational region up to a frequency of 3600 cm^{-1} , and that has its absolute maximum at a frequency of ~3500 cm^{-1} [24]. In addition, for a solution containing 25 μM SDS and up to a few mM of NaCl, the Debye screening length is long and the SFG signal originates to a large part from regions deeper down in the solution, leading to a phase distortion that enhances the negative Im($\chi^{(2)}$) signal at frequencies >3300 cm^{-1}. For NaCl concentrations >25 mM, the Im($\chi^{(2)}$) spectrum is positive at all frequencies in the OH stretch vibrational region, because the phase distortion has vanished and the surface density of DS^- ions has increased, as will be discussed later. For a solution of SDS at its critical micelle concentration (CMC) of 8 mM, the

Im($\square^{(2)}$) spectrum is also positive at all OH stretch frequencies (figure SI 2), as was also found in Ref. 26, because at this concentration the surface density of DS^- is high and the Debye screening length is short."

Reviewer comment

There is a dramatic change in the shape between no salt and + 10 mM NaCl. How do the authors rationalize this? It does not appear as a standard change in the DL-contribution, which as stated by the authors would appear in an intenistiy modulation but no change in the spectral shape. It does not seem to be something that can be explained by interferences either, since the Im Chi2 intensity decreases instead of increasing by adding 10 mM NaCl.

Author reply

The decrease of the positive contribution to the Im($\square^{(2)}$) spectrum going from 0 mM NaCl to 10 mM NaCl is largely due to screening of the electric field exerted by the DS^- ions by the added Na^+ ions. As a result, the contribution of the Im($\square^{(3)}$) spectrum of the diffuse layer to the observed Im($\square^{(2)}$) response will be smaller for a solution with 25 □□ SDS and 10 mM NaCl than for a solution with 25 □□ SDS and 0 mM NaCl. As pointed out in the reply to the previous comment, at these low (□M) SDS and (mM) NaCl concentrations, the observed Im($\square^{(2)}$) spectrum still shows similarity with that of pure H_2O that is negative up to frequencies of 3600 cm^{-1}, and that has its absolute maximum at a frequency of ~3500 cm^{-1}. This negative signal will become relatively more important going from 0 to 10 mM NaCl due to the decrease of the contribution of the Im($\square^{(3)}$) spectrum of the diffuse layer. In addition, the phase distortion of the signal will decrease going from 0 to 10 mM of added NaCl, which in fact leads to a decrease of the negative signal at at frequencies >3300 cm^{-1}. All these contributions have a different spectral dependence, leading to a strong dependence of the spectral shape of the observed Im($\square^{(2)}$) response on the NaCl concentration up to a concentration of ~25 mM. Above this concentration, the phase distortion has vanished and the DS^- surface density has become sufficiently high to let the observed Im($\square^{(2)}$) response be completely dominated by the Im($\square^{(3)}$) spectrum of the diffuse layer.

Action taken

This point is also addressed in the extended description of the Im($\square^{(2)}$) spectrum observed at low NaCl concentrations.

Reviewer 2

We thank the reviewer for again carefully reading our manuscript and for pointing out that we misunderstood one of the points of his/her/their report. Below we will address this point.

Reviewer comment

Sengupta et al made revisions according to the comments of this reviewer, and the paper is now much more readable. Most of their responses are satisfactory, but they completely misunderstood one of my comments.

In my second comment, I encouraged them to "clearly write the phase of the SDS monolayer in this study." In a surface tension–area isotherm of a monolayer, as the area per molecule is reduced, the monolayer adopts gas, liquid-expanded (LE), liquid-condensed (LC), condensed/solid (C) phases before

collapsing from the interface. I supposed that the monolayer examined in this study was the gas phase in this sense.

The authors need to provide relevant discussion on the phase in the above-mentioned meaning.

Author reply

We apologize for misunderstanding the comment made by the reviewer. For a DS^- layer the surface density and phase of the surface layer cannot be controlled in the same manner as for a Langmuir layer, i.e. by compressing the surface. The DS^- layer is not a Langmuir layer but a Gibbs layer. For a Gibbs layer, the surface density is fully determined by the equilibrium with the bulk solution. Hence, a reduction of the surface area will not lead to an increase of the surface density, but rather to surfactant ions entering the bulk, conserving the surface density.

In our study we observe a strong increase in the surface density of DS^-, following the addition of NaCl to the solution, due to screening by the added ions of the Coulomb repulsion between the negatively charged headgroups of the DS^- ions. The surface density of DS^- gets then further enhanced by the favorable van der Waals interaction of the hydrophobic tails of the DSsurfactants, which is somewhat reminiscent of the liquid condensed phase observed for Langmuir films. This means that the phase of the DS^- layer can be characterized as gas phase at low NaCl concentrations, and as liquid condensed phase at high NaCl concentrations.

Action taken

We added a few sentences explaining the phase of the SDS surface layer at different NaCl concentrations, on page 6 of the revised manuscript:
"We thus find that the addition of NaCl induces a transition from a gas phase DS^- -surface layer to a liquid-condensed phase surface layer. Such a transition is reminiscent of the behavior observed in surface tension isotherms of Langmuir monolayers, with the difference that for Langmuir monolayers the change in phase is induced by physical compression of the surface[32]."
